# Focusing the amyloid cascade hypothesis on N-truncated Abeta peptides as drug targets against Alzheimer’s disease

**DOI:** 10.1007/s00401-014-1287-x

**Published:** 2014-05-07

**Authors:** Thomas A. Bayer, Oliver Wirths

**Affiliations:** Division of Molecular Psychiatry, Georg-August-University Goettingen, University Medicine Goettingen, Von-Siebold-Strasse 5, 37075 Göttingen, Germany

**Keywords:** Pyroglutamate, Transgenic mouse model, Intraneuronal Abeta, Post-translational modification, Immunotherapy, Aβ_4–X_ oligomer

## Abstract

Although N-truncated Aβ variants are known to be the main constituent of amyloid plaques in the brains of patients with Alzheimer’s disease, their potential as targets for pharmacological intervention has only recently been investigated. In the last few years, the Alzheimer field has experienced a paradigm shift with the ever increasing understanding that targeting amyloid plaques has not led to a successful immunotherapy. On the other hand, there can be no doubt that the amyloid cascade hypothesis is central to the etiology of Alzheimer’s disease, raising the question as to why it is apparently failing to translate into the clinic. In this review, we aim to refocus the amyloid hypothesis integrating N-truncated Aβ peptides based on mounting evidence that they may represent better targets than full-length Aβ. In addition to Aβ peptides starting with an Asp at position 1, a variety of different N-truncated Aβ peptides have been identified starting with amino residue Ala-2, pyroglutamylated Glu-3, Phe-4, Arg-5, His-6, Asp-7, Ser-8, Gly-9, Tyr-10 and pyroglutamylated Glu-11. Certain forms of N-truncated species are better correlates for early pathological changes found pre-symptomatically more often than others. There is also evidence that, together with full-length Aβ, they might be physiologically detectable and are naturally secreted by neurons. Others are known to form soluble aggregates, which have neurotoxic properties in transgenic mouse models. It has been clearly demonstrated by several groups that some N-truncated Aβs dominate full-length Aβ in the brains of Alzheimer’s patients. We try to address which of the N-truncated variants may be promising therapeutic targets and which enzymes might be involved in the generation of these peptides

## Introduction

Alzheimer’s disease (AD) is a progressive neurodegenerative disorder characterized by the presence of extracellular amyloid plaques composed of amyloid-β (Aβ) surrounded by dystrophic neurites and neurofibrillary tangles. The discovery that certain early-onset familial forms of AD may be caused by an enhanced production of Aβ peptides, led to the hypothesis that amyloidogenic Aβ is intimately involved in the AD pathogenic process [88]. Aβ is derived by proteolytic cleavage of the β-amyloid precursor protein (APP) [89].

## Full-length Aβ is a physiological peptide with a role in long-term depression

As early as 1992, Haass and colleagues [30] reported the unexpected identification of full-length Aβ and the p3 fragment in media from cultures of primary cells and APP-transfected cell lines grown under normal conditions. In addition, using in vivo micro-dialysis in mice, Kang et al. [43] found that the amount of Aβ in interstitial fluid (ISF) correlated with wakefulness. ISF Aβ was assessed in Tg2576 mice at 3 months of age, several months earlier than initial deposition of Aβ. They found diurnal variation of ISF Aβ levels with significant increases (+75 %) during the dark period compared to the light period. Despite fluctuations in ISF, total tissue hippocampus homogenates levels of Aβ, full-length APP, APP C-terminal fragments, Aβ_1–40_ and Aβ_1–42_ were not significantly altered between dark and light periods. This indicates that the pool of ISF Aβ is likely to be regulated independently from total intracellular and membrane-associated Aβ. The amount of ISF Aβ also significantly increased during acute sleep deprivation and during infusion of orexin, a neurotransmitter regulating arousal and wakefulness, but decreased with infusion of an orexin receptor antagonist. Moreover, cerebrospinal fluid (CSF) levels of Aβ were studied in ten young healthy male volunteers via lumbar catheters over a 33 h period and illustrated clear evidence of diurnal fluctuation of Aβ in the CSF. Aβ levels increased throughout the day with a peak in the evening that decreased overnight. The group also reported that the APPswe/PS1ΔE9 mouse model of AD showed normal sleep–wake cycle and diurnal fluctuation in ISF Aβ in the brain before Aβ plaque formation [75].

A physiological effect of the observed diurnal variation of ISF Aβ could be the known overall increase in synaptic strength during the day and synaptic depression during periods of sleep [20, 24, 100]. These findings are corroborated by results showing that neuronal activity modulates the formation and secretion of Aβ peptides in hippocampal slice cultures bearing neurons that overexpress APP. In addition, Aβ depressed excitatory synaptic transmission in neurons expressing APP, as well as nearby neurons that did not, leading to the assumption that activity-dependent modulation of endogenous Aβ production may normally participate in a negative feedback loop that could keep neuronal hyperactivity under control [42].

Long-term depression (LTD) represents an activity-dependent reduction in the efficacy of neuronal synapses and has been described in a variety of neurons. It has been shown that Aβ is capable of regulating the amount of surface NMDA-type glutamate receptors [92]. In addition, several parallels between LTD and Aβ-induced synaptic changes have been described. Aβ-induced synaptic depression partially mimicked metabotropic glutamate receptor LTD synaptic transmission. It has been hypothesized that this could be a normal physiological role of full-length Aβ [36]. Recent results suggest that conformational changes of the NMDA receptor (NMDAR), and not ion flow through the channel, are required for Aβ to produce synaptic depression and a switch in NMDAR composition [45].

It has been suggested that N-terminally truncated Aβ_5–x_ peptides are preferentially formed by an alternative cleavage of APP involving caspase activity [67]. These N-truncations were detected in Aβ deposits of sporadic and familial AD [29, 62, 63, 72, 90, 94]. Using mass spectrometry and Western blot analysis of sporadic AD and familial AD cases (M146V *PS1* or KM670/671NL *APP*), Aβ_5–40/42_ was one of the detected N-truncated species. Regarding transgenic mouse lines, mass spectrometry of immunoprecipitated Aβ peptides also provided evidence of the presence of Aβ_5–42_, e.g. in APP/PS1KI mice [11]. Our group detected Aβ_5–42_ peptides in the 5XFAD mouse model [110] and using immunohistochemistry in APP/PS1KI, 5XFAD and 3xTG transgenic mouse models [29]. Interestingly, neither 5XFAD nor APP/PS1KI showed any evidence for intraneuronal Aβ_5–x_, which is in good agreement with the observation that this peptide is readily secreted [31]. One should note that all of the above-mentioned transgenic mouse models express the Swedish APP mutant prone to BACE cleavage at Asp-1 of Aβ. It is therefore unclear whether or not the Arg-5 truncation may possess neurotoxic properties, a matter of concern for therapeutic strategies involving BACE inhibitor treatment.

## Potential enzymatic activities leading to N-terminal truncations

**Fig. 1:**
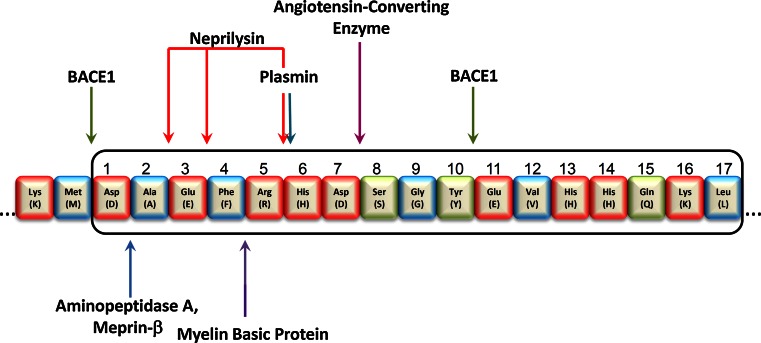
Sequence of the first 17 amino acids of the N-terminus of human Aβ is shown in three-letter and one-letter-code. Amino acids (*AA*) with charged polar side-chains are shown in *red*, AA with uncharged polar side-chains in *green* and hydrophobic non-polar AA in *blue*. The cleavage sites of enzymes involved in the degradation of full-length and potential generation of N-truncated Aβ peptides are indicated

The precise enzymatic activities leading to the generation of the diverse N-terminally truncated Aβ peptides are in most cases not known in detail; however, several candidates have been proposed (Fig. 1; Table 1).

**Table 1:** Overview of identified proteases and cleavage sites possibly involved in N-truncated Aβ generation

Protease	Cleavage site	Potential Aβ peptide	References
BACE1	Met (−1) ↓ Asp(1)	Aβ_1–x_	[98]
	Tyr (10) ↓ Glu (11)	Aβ_11–x_, Aβ_pE11–x_	
Aminopeptidase A	Asp (1) ↓ Ala (2)	Aβ_2–x_	[91]
Meprin-β	Asp (1) ↓ Ala (2)	Aβ_2–x_	[6]
Neprilysin	Ala (2) ↓ Glu (3)	Aβ_3–x_, Aβ_pE3–x_	[35, 47]
	Glu (3) ↓ Phe (4)	Aβ_4–x_	
	Arg (5) ↓ His (6)	Aβ_6–x_	
Myelin basic protein	Phe (4) ↓ Arg (5)	Aβ_5–x_	[52]
Angiotensin-converting enzyme	Asp (7) ↓ Ser (8)	Aβ_8–x_	[37]
Plasmin	Arg (5) ↓ His (6)	Aβ_6–x_	[97]

Aβ molecules secreted by MDCK cells exhibit extensive amino-terminal heterogeneity with >80 % of molecules containing an amino-terminus at the Arg-5 residue and only a minority of fragments initiating at Asp-1 [31]. In contrast to the results obtained in cells expressing wild-type APP, the cells expressing APP-695swe showed that the majority of Aβ peptides began at Asp-1, while only ~10 % began at Arg-5. The results indicated that Asp-1 is a preferred site for the β-secretase cleavage of APP-695swe [54].

N-truncation of Aβ_3–40_ and Aβ_5–40_ is facilitated by reduced endocytosis of APP in vitro, a requirement for BACE cleavage [12]. The generation of Aβ was analyzed in human embryonic kidney (HEK) 293 cell lines stably expressing wild type and non-internalizing mutants of human APP [12]. APP lacking the entire cytoplasmic domain or with both tyrosine residues of the motif GYENPTY mutated to Ala showed at least fivefold reduced endocytosis. In these cell lines, the production of Aβ_1–40_ was substantially reduced but accompanied by the appearance of two prominent alternative Aβ peptides differing at the amino termini which were identified as Aβ_3–40_ and Aβ_5–40_.

Portelius et al. [73] studied the Chinese hamster ovary cell line 7PA2 stably transfected with the 751 amino acid APP isoform harboring mutant V717F. Treatment of the cells with a BACE1 inhibitor decreased the abundance of the Aβ monomer band and resulted in lower levels of Aβ_1–40_, Aβ_1–42_ and secreted APP. Western blot bands thought to represent oligomers of Aβ increased in response to BACE1 inhibition. This increase was paralleled by the emergence of N-terminally truncated Aβ_5–40_ in particular. Treatment of cell cultures and dogs with BACE1 inhibitors significantly reduced Aβ peptides starting at Asp-1, while amino-terminally truncated variants such as Aβ_5–40_ increased [59, 83, 94]. Based on data from treatment of human neuronal and non-neuronal cells expressing wild-type APP with inhibitors of BACE and α-secretase in vitro, it has been proposed that Aβ_5–40/42_ might be derived from alternative β-cleavage of APP by α-secretase-like protease(s) [94]. One has to consider though that the expression of a mutation within the *APP* gene and/or the cell types used can influence the variant and quality of the N-terminally truncated Aβ.

Incubation of HEK293 cells overexpressing APP containing the Swedish mutation with the aminopeptidase inhibitor amastatin revealed significantly increased levels of full-length Aβ in the supernatant. This led to the identification of aminopeptidase A as a candidate enzyme cleaving the N-terminal Asp-1 residue [91]. Very recently, meprin-β has been proposed as another enzyme with the ability to process Aβ peptides [6]. It has been demonstrated that this enzyme cleaves full-length APP in a β-secretase manner, leading to the generation of Aβ_2–x_ peptides, which have been previously described in AD patients [56, 104]. One of the major Aβ-cleaving proteases is the zinc-metalloprotease neutral endopeptidase or neprilysin (NEP) [38]. High-performance liquid chromatography in combination with mass spectrometry analysis identified several cleavage sites when Aβ_1–40_ peptides were incubated with NEP. Among other truncations, NEP generates N-terminal truncated Aβ peptides by cleavage between Arg-2 and Glu-3 or between Glu-3 and Phe-4 but leaves full-length APP unaffected [35, 47]. In vitro experiments have demonstrated that exposure of cultured primary neurons to aggregated full-length Aβ leads to increased mRNA-levels of tissue plasminogen activator (tPA) and urokinase-type plasminogen activator (uPA), implying a role for the plasmin system in Aβ clearance. It has been shown that purified plasmin degrades Aβ with physiologically relevant efficiency, leading to the identification of different cleavage sites, e.g. after Arg-5, which is consistent with the known specificity of plasmin to cleave after basic amino acids [96, 97]. Genetic studies have established a relationship between angiotensin-converting enzyme (ACE) and AD [21]. Hu and colleagues [37] provided the first evidence that ACE is able to significantly counteract the aggregation, deposition and cytotoxicity of Aβ in vitro by cleavage of Aβ at Asp-7. Purified myelin basic protein (MBP) is another candidate protein that possesses endogenous serine protease activity and that, at least in vitro, has been demonstrated to degrade Aβ peptides. Mass spectrometry identified several cleavage sites in fibrillar and soluble Aβ42 preparations, including between Phe-4 and Arg-5 in the N-terminus of the Aβ-sequence [52]. Finally, the major protease responsible for the liberation of Aβ_1–x_ peptides in AD, BACE 1, is also capable of cleaving between Tyr-10 and Glu-11, leading to the release of Aβ_11–x_ peptides [98]. The identification of this β′-cleavage site matched previous observations in primary neurons and cell lines suggesting that many of the Aβ_x–40/x–42_ peptides start with Glu-11 [95, 103]. Whereas N-terminally truncated and post-translationally modified Aβ_pE11–42_ peptides have been demonstrated predominantly in mature plaque cores in AD brains, both unmodified Aβ_11–40_, as well as Aβ_pE11–40_ peptides have been detected in vascular deposits by immunohistochemistry [53].

## N-truncated Aβ is neurotoxic in vitro

Pike et al. [71] demonstrated that N-terminal deletions are neurotoxic. Interestingly, the N-truncated Aβ_x–40_ peptides exhibited an enhanced neurotoxicity in vitro, while no difference was reported between full-length Aβ_1–42_ and the other truncated Aβ_x–42_ peptides. The authors compared the aggregation characteristics and biophysical properties of Aβ starting with Asp-1, Phe-4, Ser-8, Val-12 and Lys-17. Peptides with N-terminal deletions exhibited enhanced peptide aggregation relative to full-length species, as quantitatively assessed by sedimentation analyses. Full-length and truncated peptides showed circular dichroism spectra consistent with predominant β-sheet conformation, fibrillar morphology under transmission electron microscopy, as well as significant toxicity in cultures of rat hippocampal neurons. The authors concluded that N-terminal deletions enhance aggregation of β-amyloid into neurotoxic, β-sheet fibrils and suggested that such peptides may initiate and/or nucleate the pathological deposition of Aβ into plaques. Others reported that pyroGlu-3 was found to be more neurotoxic as compared to full-length Aβ [79]. In addition, it has been demonstrated that irrespective of the C-terminus of Aβ, i.e., Aβ40 or 42, pyroGlu-3 modified Aβ peptides displayed dramatically accelerated initial formation of aggregates compared to unmodified full-length Aβ. The accelerated seed formation was accompanied by a change in the oligomerization kinetics [85]. The N-terminal pyroGlu-3 and pyroGlu-11 modifications in comparison to their non-pyroglutaminylated counterparts Glu-3 and Glu-11 or Asp-1 (only Aβ_x–40_ was investigated), revealed a decrease of solubility in the physiological pH range which was accompanied by an increase in hydrophobicity [87].

Nussbaum et al. [69] reported that Aβ_pE3–42_ and Aβ_1–42_ form metastable, cytotoxic, hybrid oligomers possessing a prion-like activity. The authors compared the cytotoxicity of the peptides in cultured neurons or glia cells and found that 12 h of 5 µM Aβ_1–42_ exposure had little effect on cell viability on wild-type or tau-knockout neurons, or wild-type glial cells. In contrast, most wild-type neurons died and detached from the substrate after exposure to 5 µM Aβ_pE3–42_ or a mixture of 5 % Aβ_pE3–42_ and 95 % Aβ_1–42_ (5 µM peptides in total). Tau-knockout neurons and wild-type glia, which express little tau protein, were resistant to Aβ_pE3–42_ and the mixture containing 5 % Aβ_pE3–42_ and 95 % Aβ_1–42_.

We have recently extended these observations showing that soluble aggregates of Aβ_4–42_ and pyroGlu Aβ_pE3–42_ have specific structural features that might carry their neurotoxic activity [7]. We demonstrated that Aβ_4–42_, Aβ_1–42_ and Aβ_pE3–42_ are unstructured in the monomeric state. However, upon heating the Aβ variants showed a high propensity to form folded structures. Monomeric Aβ_4–42_ and Aβ_pE3–42_ were rapidly converted to soluble aggregated species, whereas Aβ_1–42_ stayed in equilibrium between monomers and soluble oligomers. The soluble aggregates were capable of converting to fibrillar aggregates with Aβ_4–42_ and Aβ_pE3–42_ showing significant thioflavin-T-reactivity already during the nucleation phase of aggregation [7]. The observation that the propensity of Aβ_4–42_ and Aβ_pE3–42_ to form aggregates is more pronounced than that of the N-terminally intact Aβ_1–42_ peptide suggests that Aβ_4–42_ and Aβ_pE3–42_ aggregation may precede Aβ_1–42_ aggregation in vivo.

Using far-UV CD spectroscopy, NMR spectroscopy and dynamic light scattering, we also have demonstrated that Aβ_4–42_ and Aβ_pE3–42_, and to a lesser extent Aβ_1–42_, had a remarkable tendency to form stable aggregates [7]. The aggregates formed by Aβ_4–42_ and Aβ_pE3–42_ were distinct in size and different from Aβ_1–42_. In addition, the fibrillar structure of Aβ aggregates was studied using transmission electron microscopy. The observation that all peptides except for Aβ_1–42_ formed clumps of fibrils pointed to the importance of the N-terminal residues pyroGlu-3 and Phe-4 for aggregate morphology [7].

## N-terminally truncated Aβ peptides in transgenic animal models of Alzheimer’s disease

In recent years, N-terminal truncated Aβ peptides have been described not only in human samples, but also in a variety of transgenic AD mouse models. A thorough analysis in the APP/PS1KI mouse model using two-dimensional gel electrophoresis in combination with mass spectrometry at different time points, revealed the presence of a variety of N-truncated Aβ species [11]. In addition to full-length Aβ_1–42_ peptides, additional spots representing Aβ_4/5–42_ or Aβ_8/9/10/11–42_ were detected as early as 2.5 months of age, followed by Aβ_2/3–42_ being detectable at 4 months of age. In the respective 2D-gels, the spot corresponding to Aβ_8/9/10/11–42_ allows no further discrimination, making assumptions about the presence of Aβ_11–42_ difficult. This is an important issue, as previous in vitro data has indicated species specificity for BACE1, which is reported to be due to an amino acid difference in the murine and human Aβ-sequence at position 10 (Tyr in human and Phe in mouse). In conditioned media of mouse primary neurons transfected with human wild-type APP, only murine Aβ_11–40_ could be recovered and only co-transfection with human BACE1 led to considerable amounts of secreted human Aβ_11–40_ [10]. This might lead to significant bias in the assessment of N-truncated Aβ variants in transgenic mouse models and the fact that most available models harbor the Swedish APP mutation favoring the generation of full-length Aβ peptides may skew results even further. In the APP/PS1KI mouse model pyroGlu-modified Aβ_3–X_ becomes detectable at 6 months and increases in abundance with aging [11]. Subsequent immunohistochemical studies using the APP/PS1KI mouse model revealed the presence of plaque-associated and intraneuronal pyroglutamate Aβ_3–x_ [8] or in spinal cord motor neurons [105]. Pyroglutamate Aβ_3–x_-positive plaques increase significantly in abundance but at the expense of plaques containing full-length-Aβ (starting with Asp-1) which show a corresponding decrease in abundance [107]. This suggests that in the parenchyma, pyroglutamate Aβ-formation might represent a later step in plaque maturation which might depend on remodeling of existing extracellular deposits. The presence of pyroglutamate Aβ deposits in transgenic mouse models has been confirmed in a variety of studies demonstrating that pyroglutamate Aβ-immunoreactivity is mainly confined to the amyloid core [23, 33, 40, 55]. In order to verify the in vivo toxicity of pyroglutamate Aβ, mouse models expressing solely the respective peptide but not the entire human APP molecule have been developed. These models made use of constructs starting with an N-terminal glutamine residue at position 3, which has been demonstrated to represent a better substrate for enzymatic conversion to pyroGlu-3 [13]. Abundant intracellular Aβ_pE3–42_, followed by subsequent loss of Aβ_pE3–42_-accumulating neurons could be demonstrated [1, 106]. This cell loss was rescued by crossing to a Tau knock-out background [69].

In order to study a potential seeding effect of Aβ_pE3–42_ on full-length Aβ in transgenic mice, Aβ_pE3–42_ expressing mice (TBA42 model) were crossed with 5XFAD mice [110]. The resulting bigenic model FAD42 was examined at 6 months of age. FAD42 mice showed an aggravated behavioral phenotype compared with the single transgenic parental 5XFAD or TBA42 lines. ELISA and plaque load measurements revealed that Aβ_pE3–x_ levels were elevated in FAD42 mice; however, no change in Aβ_x–42_ or other Aβ isoforms was detected by ELISA or mass spectrometry. As Aβ_1–42_ is the most abundant peptide in 5XFAD and FAD mice, these observations point to a drastic effect of Aβ_pE3–42_.

Mass spectrometric analysis of 5XFAD mouse brain following immunoprecipitation with pan-Aβ or pyroGlu-specific antibodies also revealed the occurrence of Aβ_1–42_, Aβ_1–40_, Aβ_pE3–40_, Aβ_pE3–42_, Aβ_3–42_, Aβ_4–42_ and Aβ_5–42_. Aβ_4–42_ was the most abundant species among the N-truncated forms, but Aβ_1–42_ clearly had the highest levels of all peptides [110]. Using NT4X-167, an antibody recognizing the N-terminus of N-truncated Aβ species with a preference for Aβ_4–x_, strong intracellular staining could be detected in young 5XFAD transgenic mice [3]. Very recently, a transgenic mouse model overexpressing Aβ_4–42_ without any mutations under the control of the murine neuron-specific Thy1-promotor has been described. These mice develop a massive age-dependent CA1 pyramidal neuron loss which correlates with the transgene expression pattern in the hippocampus. In addition, age-dependent spatial reference memory deficits were detected using the Morris water maze paradigm, underscoring the in vivo toxicity of Aβ_4–42_ peptides [7].

However, in relative amounts, N-terminally truncated Aβ peptides, and in particular Aβ_pE3–42_, in transgenic mouse models are much less abundant compared to human brain samples [76, 84]. In very old Tg2576 mice (21–23 months), only 5 % of the total insoluble Aβ is N-terminally truncated, whereas the corresponding percentage in human brain is ~70–85 % [44]. The relative solubility of human and APP transgenic mouse amyloid is strikingly different. Whereas, e.g. amyloid cores in Tg2576 and APP23 mice are completely soluble in SDS solutions with EDTA, human amyloid deposits are much more stable and do not dissociate in the presence of ionic or nonionic detergents or strong denaturing agents like guanidine hydrochloride. Therefore, the increased solubility of transgenic mouse amyloid might be directly related to the relative absence of N-terminal truncations and other post-translational modifications [41].

Together with Glu-3 of Aβ, the N-terminus of monocyte chemoattractant protein 1 (CCL2 or MCP-1) is modified to a pyroglutamate residue protecting against degradation in vivo. Cynis et al. [14] showed that the pyroGlu-formation of MCP-1 depends on glutaminyl cyclase (QC) activity. The same group has also provided strong evidence that Glu-3 of Aβ is pyroglutamated by QC [86]. Genetic ablation of the glutaminyl cyclase iso-enzymes QC or isoQC revealed a major role of isoQC for pyroGlu-MCP-1 formation and monocyte infiltration [14]. As neuroinflammatory processes around amyloid plaques represent a major hallmark of AD, it is likely that glial activation leads to enhanced QC activity and subsequent pyroGlu-3 formation in AD plaques. In line with that notion, we have observed that during plaque maturation the amount of Aβ peptides with intact N-terminus starting with Asp-1 declines whilst pyroGlu-3 increases [107].

While it is clear that transgenic mice expressing only N-truncated Aβ peptides Aβ_pE3–42_ and Aβ_4–42_ do develop massive neuron loss in CA1 [1, 7], the mechanisms of cell death have not been elucidated. Palop and Mucke [70] discussed that AD is associated with cognitive decline and increased incidence of seizures. Sporadic cases are known to exhibit seizure activity, as well as many pedigrees with autosomal dominant early-onset AD, including those with mutations in presenilin-1, presenilin-2, or APP, or with duplications of wild-type APP. Moreover, high levels of Aβ in the brain of APP transgenic mouse models can cause epileptiform activity.

## N-truncated Aβ peptides appear during Alzheimer progression

Due to variations in the methods and tools used to extract and identify different pools of Aβ, drawing conclusions on the exact levels of the various N-truncated Aβ variants is challenging. Evaluation of data is difficult as far as the exact levels of different Aβ peptides in post-mortem brains are concerned. Many factors can influence an analysis ranging from antibody specificities and sensitivities in applications like immunostaining, Western blotting or immunoprecipitation, as well as extraction protocols and brain areas studied. Most consistently, there is general agreement that plaque-born peptides harbor high amounts of N-truncated Aβ especially Phe-4, but also pyroGlu-3 and pyroGlu-11. In presymptomatic AD cases, Phe-4 seems to be the N-truncated variant most consistently reported. Besides plaque-associated Aβ, intraneuronal Aβ can be N-truncated in AD brain [27]. Immunohistochemical studies in cases with Down syndrome demonstrated plaque-Aβ starting at Asp-1 or pyroGlu-3 [48]. A transient accumulation of intraneuronal Aβ_x–42_ was also evident [66]. Analyzing FAD patients, Ancolio and colleagues [2] firstly showed a selective and drastic increase of N-truncated Aβ_x–42_ species triggered by the mutation *APP* V715M. In contrast to the N-terminus, there is common agreement that plaque-associated Aβ peptides mainly terminate at position 42 with Ala-42.

In the following paragraphs, we endeavor to shed light on what is known regarding the role of N-terminal truncated Aβ peptides in AD.

In 1985, ragged Aβ peptides were described to precipitate in AD plaques, including a major species beginning with phenylalanine at position 4 of Aβ (Phe-4; Aβ_4–x_) [58]. A majority of 64 % of the peptides in amyloid plaques of the two sporadic AD cases and of 45 % in the patients with Down syndrome studied started with a Phe-4 residue. At the same time, Glenner and Wong [26] demonstrated full-length Aβ beginning with Asp-1 to be the main species detected in cerebrovascular deposits. A scheme of the amino acid residue numbering of N-terminal Aβ is shown in Fig. 1.

Miller et al. [62] compared the peptide compositions of the cerebrovascular and senile plaque core amyloid deposits in AD. Matrix-assisted, laser-desorption-time-of-flight (MALDI-TOF) mass spectrometry of plaque-Aβ revealed an array of peptides ending with Ala-42 of that sequence, while cerebrovascular Aβ began with Arg-1 ending at Val-40. They verified that Phe-4 is the main component in plaques, but cautioned that their MALDI-TOF spectral data suggests the presence of two pyroglutamyl amino termini (pyroGlu-3 and pyroGlu-11) that might escape detection by other methods. Other N-termini reported were Asp-1, Ala-2, Arg-5, Asp-7, Ser-8, Gly-9.

Surface-enhanced laser desorption/ionization mass spectrometry was performed comparing AD and vascular dementia patients [51]. In AD, the authors found Aβ starting with Asp-1, Ala-2, pyroGlu-3, Phe-4 and Arg-5 in senile plaque extractions with Phe-4 to be the most prevalent one.

The presence of pyroGlu-3 peptides as an important component of plaque depositions in patients with AD was further substantiated [77, 80]. MALDI-TOF mass spectrometry of Aβ peptides isolated from sporadic and familial AD (*APP* V717I and several *PS1* mutations) brains indicated that besides full-length Aβ_1–40/42_; pyroglutamylated Aβ_3–42_ (Aβ_pE3–42_) and Aβ_pE11–42_ as well as Aβ_4–42_ were detected in these cases [78]. Analysis of sporadic and familial AD cases by electrospray–ionization mass spectrometry even showed that Aβ_11–42_/Aβ_pE11–42_ represent the second most abundant species following Aβ_1–40_ [68]. Further analysis of FAD cases revealed that N-terminally truncated Aβ peptide species ending at residues 42 and 43 are the main Aβ peptides deposited in brain parenchyma in association with the *PS1* V261I mutation. MALDI-TOF mass spectrometry following immunoprecipitation using a mixture of Aβ antibodies showed that most intense signals corresponded to pyroGlu-11, pyroGlu-3, but also non-pyroglutamylated Glu-3 peptides, whereas the signals corresponding to Glu-11 and Asp-1 were less intense [63].

The Aβ isoform pattern was studied in the cerebellum, cortex and hippocampus in AD, including subjects with mutations in *PS1* (M146V) or *APP* (KM670/671NL) genes, sporadic AD subjects and non-demented controls [72]. Using immunoprecipitation in combination with mass spectrometric analysis, the dominating Aβ isoforms in the three different brain regions analyzed from control, sporadic and familial AD were described as Aβ_1–42_, Aβ_pE3–42_, Aβ_4–42_ and Aβ_1–40_, with Aβ_1–42_ and Aβ_4–42_ being the dominant isoforms in hippocampus and cortex in all groups analyzed [72].

The question whether N-truncations of Aβ are a post-mortem artefact or might even precede the symptomatology of AD was addressed by Sergeant and co-workers [90]. They have adapted a proteomic method in combination with Western blotting and mass spectrometry for the characterization of insoluble Aβ extracted in formic acid. Full-length Aβ peptides represented 37 % of all Aβ species, while 17 % corresponded to N-truncated species starting at residues Phe-4, Arg-5 and 20 % with Ser-8, Gly-9 and Tyr-10. They also demonstrated that the first stage of amyloid deposition in non-demented individuals comprise N-terminal truncated variants starting at positions 4-, 5-, 8- and 9–42, or with a pyroglutamyl residue at position 3. At this stage, Aβ oligomers were exclusively made of Aβ_x–42_ species.

N-terminal truncations of Aβ, especially pyroGlu-3 were reported to be more frequently found in plaques of sporadic AD cases as compared to the PS2APP mouse model [28].

CNS and the cerebrospinal fluid from APP23 transgenic mice were assessed using one- and two-dimensional gel electrophoresis, immunoblotting and mass spectrometry [84]. Significant differences between APP23 mice and brain samples from sporadic AD cases (Braak stage V–VI) were observed in their relative abundance of specific variants of Aβ peptides, such as pyroGlu-3, Aβ_1–42_ and N-terminally truncated Aβ_2/3–42_.

In a recent report, phosphorylated Aβ at Ser-8 (pSer-8) and pyroGlu-3 in soluble, dispersible, membrane-associated and plaque-associated amyloid-β aggregates in brains from 21 cases with symptomatic AD, 33 pathologically preclinical AD cases, and 20 control cases was compared [74]. Plaques containing pSer-8 were detected in all symptomatic cases with AD, but only in a few non-demented control subjects. The deposition occurred in a hierarchical sequence with pyroGlu-3 appearing early in the amyloid cascade corroborating earlier findings in preclinical AD cases [90].

Moore et al. [64] employed sequential pull-down with antibodies Ab9 (epitope Aβ_1–16_) and 4G8 followed by mass spectrometry using brain samples from the prefrontal cortex. 16 brains from AD, eight brains from subjects without clinical evidence of dementia and seven brains with rare or no AD lesions from elderly individuals without clinical evidence of a neurological illness were studied. In the membrane-associated SDS extracted lysates Aβ_1–42_, Aβ_4–42_ and Aβ_1–40_ were the most prevalent peptides. In plaque-associated formic acid lysates the spectrum became more diverse. The two pyroglutamylated peptides Aβ_pE3–42_ and Aβ_pE11–42_ were showing up, albeit at low levels compared to Aβ_4–42_, Aβ_8–40_, Aβ_8–42_, Aβ_9–40_ and the full-length Aβ_1–42_, Aβ_1–43_ and Aβ_1–40_. The presymptomatic group revealed elevated Aβ_4–42_, Aβ_pE3–42_ and Aβ_1–42_ levels compared to the control group.

**Fig. 2:**
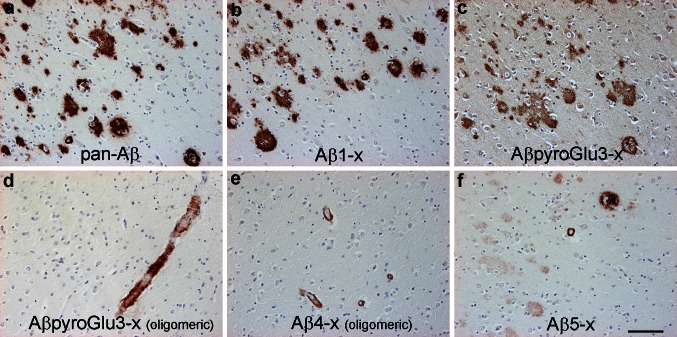
Comparative immunostaining against intact N-terminus and most prevalent N-truncated Aβ peptides Aβ_pE3–X_, Aβ_4–X_ and Aβ_5–X_ in the brain of patients with sporadic Alzheimer’s disease. Staining was performed with antibodies 4G8 (**a** epitope Aβ_17–24_), IC16 (**b** against Aβ_1–x_; gift by Sascha Weggen [39]), 2–48 (**c** against Aβ_pE3–X_; Synaptic Systems [107]), 9D5 (**d** against oligomeric Aβ_pE3–X_; Synaptic Systems [108]), NT4X-167 (**e** against oligomeric Aβ_4–X_; [3]) and AB5-3 (**f** against Aβ_5–X_; PSL Heidelberg [29]). *Scale bar* 100 μm

A comparison of antibody staining profiles in the brain of a patient with sporadic AD against Asp-1, pyroGlu-3 (pan- and oligomer-specific), Phe-4- and Arg-5 is shown in Fig. 2. Antibodies against pan-Aβ, Asp-1 and pan-pyroGlu-3 stained amyloid plaques very strongly, whereas the oligomer-specific antibodies (9D5 against pyroGlu-3 and NT4X-167 against Phe-4) labeled predominantly cerebrovascular deposits. AB5-3 against position 5 of Aβ detected both hallmarks with a tendency towards stronger labeling of cerebrovascular deposits. The cerebrovascular localization indicates that these N-truncated Aβ peptides are forming preferentially soluble aggregates with a reduced tendency to aggregate in plaques.

## Aβ as target for immunotherapy

In 1999, Schenk et al. [82] pioneered the AD field by introducing immunization as a causal therapeutic option. They immunized transgenic APP mice with pre-aggregated synthetic Aβ_1–42_, either before or after onset of plaque deposition. Immunization of young animals essentially prevented the development of plaque formation and astrogliosis. Treatment of older animals also markedly reduced the extent and progression of these AD-like neuropathologies. These results implied that immunization with pre-aggregated Aβ_1–42_ may be effective in preventing and treating AD. Moreover, vaccination with Aβ not only reduced plaque load, but also protected transgenic mice from the learning and age-related memory deficits [65]. Several mechanisms have been suggested since then for the significant therapeutic effects of immunotherapy in AD mouse models, which will be discussed below.

### Clearing plaque Aβ

Antibodies may act catalytically to dissolve preformed Aβ aggregates or prevent Aβ aggregation [93]. In this case, the antibody pool might also be neutralized by amyloid plaques due to plaque binding, leading to weakened efficacy. The phase II clinical trial with AD patients using pre-aggregated synthetic Aβ_1–42_ for active immunization was very instructive, despite the fact that it had to be stopped due to unexpected side effects with 6 % of AD subjects (18 of 300) developing serious brain inflammation resembling meningoencephalitis [25]. Although immunization with Aβ_1–42_ resulted in clearance of amyloid plaques in patients with AD, the clearance did not prevent progressive cognitive decline [34]. While these observations clearly showed that peripheral antibodies against Aβ do have an effect on CNS molecules like deposited amyloid peptides, simple plaque removal is not sufficient to rescue AD memory decline. Antibodies targeting plaques could even have a noxious effect by solubilizing fibrillar and innocuous Aβ [5, 32].

Bapineuzumab was the first humanized antibody in clinical trials. However, in double-blinded, randomized, placebo-controlled phase III trials involving more than 2,000 patients with mild-to-moderate AD, bapineuzumab did not improve clinical outcomes [81]. The crystal structure of a bapineuzumab Fab–Aβ peptide complex revealed that it captured Aβ in a monomeric helical conformation at the N-terminus [61]. The authors used microscale thermophoresis to demonstrate that the Fab binds soluble Aβ_1–40_ with a KD of 89 (±9) nM. They concluded that the crystal structure explains the antibody’s selectivity for monomeric Aβ species and that it cannot recognize N-terminally modified or truncated Aβ peptides.

### Clearance by microglia

Microglia clearance of Aβ is another mechanism that has recently been proposed as being important in an immunotherapy approach [101]. Intracranial administration of anti-Aβ antibodies into frontal cortex and hippocampus of Tg2576 APP transgenic mice resulted in clearance of compact amyloid deposits and is associated with microglial activation [102]. This might lead to subsequent phagocytosis via an interaction of the Fc receptor on microglia cells with the Fc part of the antibody bound to Aβ [4]. In contrast, using Fc receptor-gamma chain knock-out mice, the effects of anti-Aβ antibodies on Aβ deposition in Tg2576 APP transgenic mice were not dependent on FcR-mediated phagocytic events [16]. Therefore, Fab fragments of therapeutic antibodies might be sufficient, as the Fc part is not required for Aβ neutralization.

### Peripheral sink hypothesis

Another therapeutic option does not require penetration of the blood–brain barrier. Chronic treatment with the monoclonal anti-Aβ antibody m266 led to increased plasma levels of Aβ and reduced amyloid plaques in the PDAPP transgenic mouse model [17]. In a follow-up study, the group reported that administration of m266 to these mice rapidly reversed memory deficits without altering brain Aβ burden [19]. They also found that an Aβ/antibody complex was present in both the plasma and the cerebrospinal fluid of m266-treated mice. The authors concluded that the observed treatment effect might be due to enhanced peripheral clearance and (or) sequestration of a soluble brain Aβ species [19]. In contrast, Yamada et al. [111] have reported that immunotherapy with m266 neutralizes intracerebral, rather than peripheral, soluble, monomic forms of Aβ.

### Soluble aggregates as possible target

Yet a further mechanism proposes the ability of certain antibodies to bind to oligomers and neutralize their synaptotoxic effects directly [46]. It has been shown that intra-cerebroventricular injection of naturally secreted human Aβ oligomers (harvested from 7PA2-conditioned medium) inhibited long-term potentiation in rat hippocampus. Injection of a monoclonal antibody to Aβ completely prevented the inhibition of long-term potentiation even after Aβ exposure. The N-termini of the naturally secreted oligomers were not described, therefore Asp-1 or N-terminally truncated forms could account for the observed effects.

## Autoantibodies against N-truncated Aβ

Using peptide microarrays, the presence of natural antibodies against Aβ in plasma samples and cerebrospinal fluid of AD patients and healthy controls aged 21–89 years was reported [9]. Antibody reactivity was most prominent against oligomeric Aβ and pyroGlu or oxidized residues. Interestingly, IgG levels specific for oligomeric preparations of Aβ_1–42_ declined with age and AD progression. In good agreement, we have observed that the levels of pyroGlu-IgM autoantibodies significantly decreased in AD patients as compared to non-demented controls [57]. In the group of mild cognitive impaired patients there was a significant positive correlation between pyroGlu-IgM autoantibodies and cognitive performance, i.e. individuals with high levels of pyroGlu-IgM autoantibodies obtained higher scores in the Mini Mental State Examination test battery.

## N-truncated Aβ as a target for immunotherapy

**Fig. 3:**
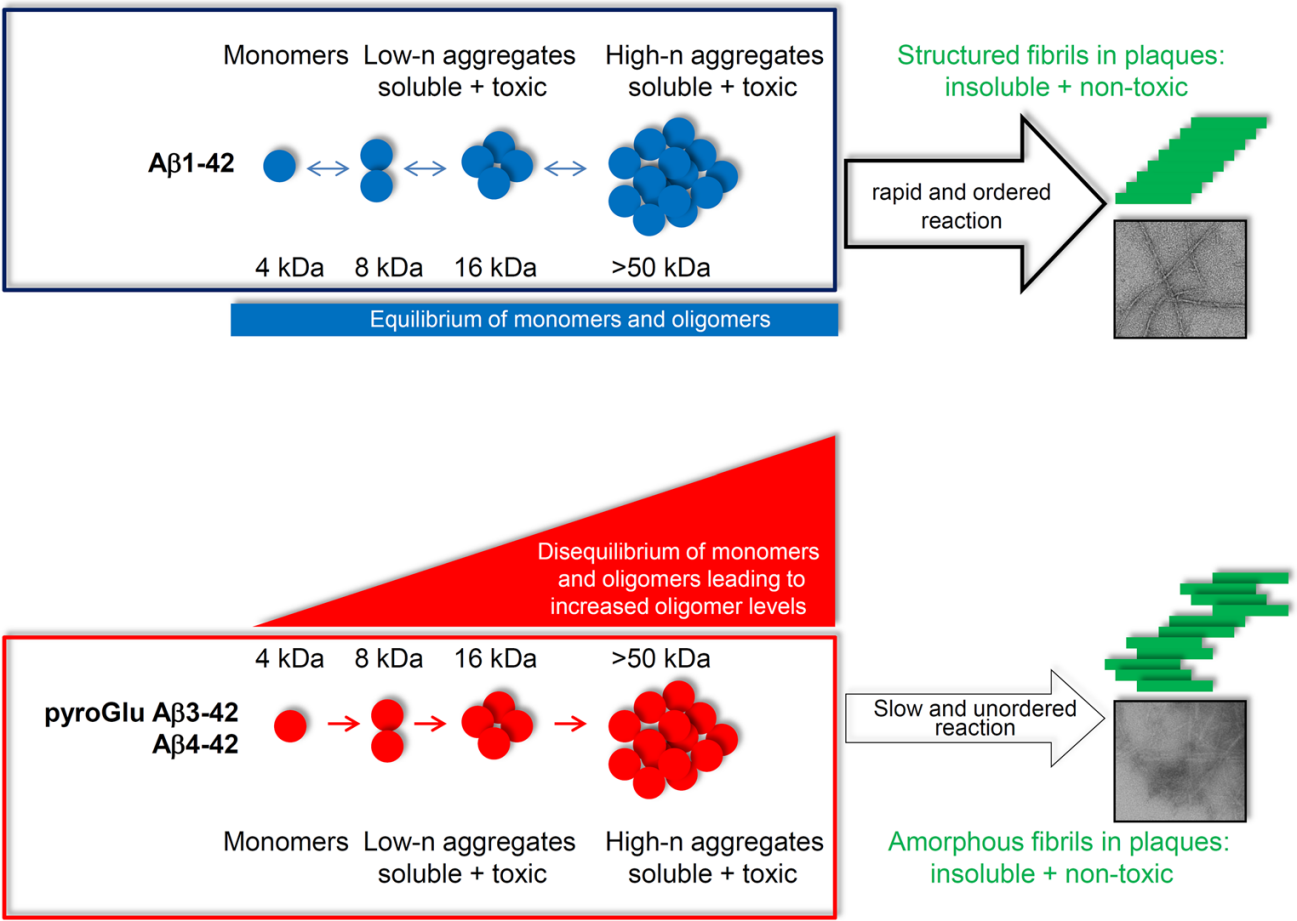
N-truncated pyroglutamate Aβ_3–42_ and Aβ_4–42_ are more toxic as compared to full-length Aβ_1–42_ due to reduced neutralization via plaque formation. *Upper graph* Monomers and low- and high-molecular weight aggregates of Aβ_1–42_ (*blue*) are in *equilibrium* and are toxic as long as they stay soluble [7]. Once high-molecular weight aggregates are formed, they rapidly react into highly ordered and insoluble, non-toxic fibrils found in plaques. Therefore, soluble low- and high-molecular weight oligomers are toxic, but can escape toxicity by forming monomers and/or fibrils. As Aβ_1–42_ is a physiological peptide, which is continuously generated also in healthy individuals, plaque formation may be one way to neutralize full-length Aβ during the prodromal stage of the disease. *Lower graph* Soluble monomers, low- and high-molecular weight aggregates of N-truncated pyroglutamate Aβ_3–42_ and Aβ_4–42_ (*red*) are in *disequilibrium* and are toxic [7]. High-molecular weight aggregates also can be neutralized by plaque formation, but with a significant slower tendency as compared to full-length Aβ, because the fibrillization process is unordered forming only amorphous fibrils. As a consequence, the level of soluble low- and high-molecular weight aggregates of N-truncated Aβ variants increase over time, thereby playing a major role in AD pathology

In contrast to Aβ_1–42_, N-truncated pyroglutamate Aβ_3–42_ and Aβ_4–42_ peptides are not produced under normal, non-disease conditions. Pyroglutamate Aβ_3–42_ and Aβ_4–42_ form soluble aggregates and are toxic in vitro and in vivo. On the basis of these empirical data, we formulated a novel hypothesis on the role of soluble aggregates of pyroglutamate Aβ_3–42_ and Aβ_4–42_ (Fig. 3).

### Pyroglutamate Aβ_3–X_ as a target

We have introduced novel conformation-specific monoclonal antibodies (9D5 and 8C4) detecting low molecular weight pyroGlu-modified Aβ oligomers [108]. The selectivity to low molecular weight (4–10 mers) pyroGlu-3 was confirmed by size exclusion chromatography and immunoblot assays. When the 9D5 antibody was added to Aβ_pE3–42_ monomers, it efficiently decreased the formation of higher aggregates, but did not interfere with the rapid formation of Aβ_1–42_ aggregates. 9D5 treatment of SH-SY5Y neuroblastoma cells abolished the toxic effects of Aβ_pE3–42_ peptides, while no beneficial effect was seen on Aβ_1–42_-induced toxicity. Passive immunization with 9D5 antibody in 4.5-month-old 5XFAD mice for 6 weeks reduced Aβ plaque load and Aβ_pE3–x_ levels [108]. This antibody labeled only a minor proportion of extracellular plaques in sporadic AD cases [99, 108].

APPswe/PS1ΔE9 transgenic mice received weekly intraperitoneal injections of an antibody against the N-terminus of pyroGlu-3 (mAb07/1). The preventative treatment protocol lingered from 5.8 to 13.8 months of age, whereas the therapeutic treatment ranged from 23 to 24.7 months of age. Passive immunization significantly reduced total plaque deposition in hippocampus and cerebellum in both treatment studies, however, insoluble Aβ levels were not affected [22].

Prior preclinical studies have shown that both active and passive immunotherapies were effective in lowering plaques in transgenic APP mice when performed as a preventative treatment; however, when performed as a therapeutic approach in aged transgenic mice, they lacked any effect on plaque levels [15, 50].

Using antibodies specific for the N-terminus of Aβ_pE3–x_, De Mattos and colleagues [18] reported that passive immunization of PDAPP mice reduced pre-existing plaques without inducing microhemorrhage in a dose-dependent manner. In an initial experiment, chronic administration of the N-terminal antibody 3D6 (the murine equivalent of bapineuzumab) significantly lowers plaque deposition when treatment was started at 9 months of age (preventative trial), but fails to alter deposition when used in a therapeutic regimen beginning at 18 months of age. Next, the novel antibody mE8 specific for the pyroGlu-modified N-terminus of Aβ_3–x_ (does not recognize full-length Aβ or unmodified Aβ_3–x_) was used for passive immunization of 23–24 month-old PDAPP mice for 3 months at a weekly intraperitoneal dose of 12.5 mg/kg. Treatment with mE8 significantly lowered Aβ42 by 30 % in the hippocampus as compared to the starting time point thus demonstrating clearance of existing Aβ deposits. The authors [18] speculated that the only mechanism of action through which Aβ_p3–x_ antibodies could lead to plaque lowering is through phagocytosis of existing plaques. It is, however, less effective at preventing Aβ42 deposition in young PDAPP mice. In contrast, the N-terminal antibody 3D6, which binds soluble and insoluble Aβ, revealed an opposite pattern of efficacy (no clearance of existing plaques and strong prevention of deposition), thereby suggesting that the major mode of action for these two antibodies is different [18]. Consistent with such a mechanism, they observed that treatment with 3D6 led to increased microglial colocalization with amyloid deposits in vivo. We have also observed that pyroGlu-3 peptides can be observed in microglia in the APP/PS1KI mouse model as an indication of phagocytotic activity [109].

### Aβ_4–x_ as a target

Although first identified in 1985 [58], Aβ_4–x_ has not received much attention as a potential therapeutic target. McLaurin et al. [60] have performed an active immunization approach in TgCRND8 transgenic mice using protofibrillar Aβ_1–42_ peptides. The mice developed robust titers against Aβ and the sera isolated from these mice stained mature, but not diffuse plaques in TgCRND8 mice. The therapeutically active antibodies were subsequently isolated and characterized. Interestingly, although protofibrillar Aβ_1–42_ was used as vaccine, beneficial effects in mice arose from antibodies selectively directed against residues 4–10 of Aβ42. These antibodies inhibited both Aβ fibrillogenesis and cytotoxicity without eliciting an inflammatory response.

We have recently generated the Aβ_4–x_-specific antibody NT4X-167 [3]. While NT4X-167 significantly rescued Aβ_4–42_ toxicity in vitro, no beneficial effect was observed against Aβ_1–42_ or Aβ_pE3–42_ toxicity. Phenylalanine at position four of Aβ was imperative for antibody specificity, because its replacement with alanine or proline completely prevented binding. Although amyloid plaques were observed using NT4X-167 in 5XFAD transgenic mice, it barely reacted with plaques in the brain of sporadic AD patients and familial cases with the Arctic, Swedish and the presenilin-1 PS1Δ Exon9 mutation. Most interestingly, Aβ_4–x_ preceded the occurrence of Aβ_pE3–x_ in the 5XFAD mouse model.

Overall, we would suggest that N-truncated pyroglutamate Aβ_3–42_ and Aβ_4–42_ peptides represent ideal therapeutic targets to fight AD for the following reasons: (1) they are produced only in diseased brain and not normal brain; (2) they aggregate quickly and irreversibly into soluble toxic oligomers; (3) only slowly react further into inert amorphous fibrils (Fig. 3); (4) are seeding aggregation of Aβ_1–40_ and Aβ_1–42_. It is worthy of note that the mechanisms discussed in this review are not exclusive and may overlap under certain circumstances. Moreover, different stages of the disease may be associated with one particular mechanism more so than the other [49].

In summary, we conclude that

- There is strong evidence that full-length Aβ peptides serve a physiological function in long-term depression and are tightly regulated during day and night in the interstitial fluid in healthy individuals.
- N-truncated Aβ variants correlate well with presymptomatic AD with Ala-2, pyrGlu-3, Phe-4, Arg-5, Ser-8 and Gly-9 often reported, but predominantly pyroGlu-3 and Phe-4.
- There is general agreement that N-truncated Aβ peptides are abundant in brains of patients with diagnosed sporadic and familial AD. Of the N-truncated variants pyroGlu-3 and Phe-4 truncations were most consistently reported.
- APP transgenic mouse models generate N-truncated Aβ peptides, albeit at quite low levels not reflecting the situation in AD brain.
- Transgenic mouse models that solely express Aβ_pE3–42_ (Glu-3 mutated to Gln-3 in order to facilitate pyroGlu-3 formation) consistently develop neuron loss and associated neurological deficits. Plaque load is low.
- The transgenic mouse model Tg_4–42_ expressing Aβ_4–42_ is the first model to harbor no mutation in the Aβ sequence and develops an age-dependent hippocampus-related reference memory deficits in the Morris water maze due to the drastic CA1 neuron loss. No plaque pathology is observed.
- Aβ_pE3–42_ and Aβ_4–42_ rapidly form soluble toxic aggregates in vitro having different biochemical properties than full-length Aβ_1–42_.
- Antibodies reacting with the N-terminus of pyroGlu-3 and Phe-4 recognize neoepitopes distinctly different from antibodies reacting with full-length Aβ peptides.
- Passive immunization with antibodies against pyroGlu-3 of transgenic mouse models demonstrated beneficial effects: no risk for microbleedings, lower pyroGlu-3 Aβ levels and reduction of pre-existing amyloid plaques.
